# Adolescent nursing consultation: an important excerpt from care provided by nurses in a Brazilian state*

**DOI:** 10.1590/1518-8345.6122.3801

**Published:** 2022-11-07

**Authors:** Hingrid Cristiane Silva Robba, Andréa Aoki Costa, Ana Cristina dos Santos Monteiro, Debora Renata Carneval, Lisabelle Mariano Rossato, Juliana Caires de Oliveira Achili Ferreira

**Affiliations:** 1 Universidade de São Paulo, Faculdade de Medicina, Hospital das Clínicas, Instituto da Criança e do Adolescente, Divisão de Enfermagem, São Paulo, SP, Brazil.; 2 Hospital Cruz Azul, Divisão de Enfermagem, São Paulo, SP, Brazil.; 3 Universidade de São Paulo, Escola de Enfermagem, Departamento de Enfermagem Materno-Infantil e Psiquiátrica, São Paulo, SP, Brazil.; 4 Universidade de São Paulo, Faculdade de Medicina, Hospital das Clínicas, Instituto da Criança e do Adolescente, Centro de Pesquisa Clínica, São Paulo, SP, Brazil.

**Keywords:** Adolescent, Adolescent Health, Adolescent Medicine, Nursing, Therapy, Patient Care Planning

## Abstract

**Objective:**

the care of adolescents with or without a chronic disease must complete, standardized and focused on individual demands and the transition process to adult care and adherence to treatment. This study aimed to characterize the care provided by nurses from the state of São Paulo who work with adolescents.

**Method:**

this is a cross-sectional and descriptive study on the care provided to adolescents by nurses in São Paulo, based on the answers to a self-administered questionnaire, available in the REDCap tool between August 2018 and October 2019.

**Results:**

participants answered 1632 questionnaires. Only 38% of nurses work with adolescents, 11.2% exclusively. Professionals were divided according to the median length of professional experience in groups A and B (≤5 years and >5 years). Drug addiction (p=0.01) and working with a multidisciplinary team (p=0.04) were significantly more reported in group B. Routine follow-up (p=0.02) and questioning about sexual or physical violence (p=0.03) were significantly more performed by professionals from group A.

**Conclusion:**

this study identified the need for a care protocol that can be replicated on a large scale and that includes the treatment and the particularities of adolescents to improve adherence and the transition into adult care.

## Introduction

Comprehensive care specific to adolescent health must consider biological, psychological, and social changes that take place during this developmental period. The transition to adulthood includes complex life characteristics and factors such as structural and functional maturation of the brain, doubts about sexual identity, pregnancy, body image and self-care responsibilities, violence, and bullying must be considered during the nursing consultation^1^ ^-^ ^2^.

Organizations need to train their professionals using participatory education and driven by social pressures, such as increased education, level of information of people and technological innovations, as well as motivation and expectation of people to participate in decisions, results, and future of the company^3^.

Adolescent medicine has recently been adopted as a specialty in high-income countries, which explains why many health professionals lack sufficient training and skills to properly work with adolescents. In low- and middle-income countries, debates regarding the training requirements for physicians and public health professionals remain incipient^4^.

Nurses are important to the health system when considering, for example, the implementation and maintenance of health policies, since these professionals play an awareness-raising role in the health system, given their intense contact with the community, contributing, directly or indirectly, to improve the quality of life of individuals^5^.

Given this scenario, the care provided by nurses and the availability of tools to be used in daily clinical practice and treatments must be characterized; regarding tools, they may be different when it comes to adolescent care, and are yet to be systematically evaluated in nurses working with this population in the state of São Paulo. The state is the richest and most populous of Brazil, having a diverse population with a large number of immigrants. São Paulo is a health reference in Brazil, providing care for a considerable percentage of patients from other states- considering the rate of adolescents (aged 10 to 19 years) in São Paulo of 5,570,389 individuals^6^. Therefore, this study aimed to characterize the care provided by nurses from São Paulo who work with adolescents.

## Method

### Type of study

This is a descriptive and observational cross-sectional study, which analyzed, based on the answers to a self-administered questionnaire, the care provided by nurses from São Paulo to adolescents.

### Study location

The state of São Paulo because it has the largest population contingent, corresponding to 21.7% of the country’s total population, and with the largest number of registered nurses among the regional nursing councils^7^ ^-^ ^8^.

### Data collection tool

The self-administered questionnaire consisted of 40 questions to characterize the profile of the participants, evaluating their experience in managing adolescents, consisting of multiple-choice or dichotomous questions (yes and no). The questionnaire’s ninth question (“Do you work with adolescents?”) functioned as an exclusion criterion, that is, if the professional answered “No,” their participation ended at that moment. The estimated length of response was approximately 15 minutes.

### Data collection

Data collection was carried out via the application of the self-administered questionnaire between August 2018 and October 2019. The electronic survey included questions related to the following topics:

1. Demographic data of professionals (gender, years of practice, country), location of professional practice (public university, private university, public clinical practice and/or private clinical practice).
2. Employment of professionals (part-time or full-time).
3. Graduate degree (*lato sensu* and/or *stricto sensu*).
4. Reason for choosing to work with adolescents.
5. Remuneration.
6. Number of adolescent patients/month followed in the last year by professionals.
7. Vaccination booklet.
8. Individualized and targeted care for the adolescent.
9. Supportive care available.
10. Program focused on the transition to adult care.
11. Use of a clinical assessment tool to screen for substance-related risks and problems in adolescents named CRAFFT, (acronym for Car; Relax; Alone; Forget; Family/Friends; Trouble) translated into Portuguese CESARE (acronym for *Carro*; *Esqueceu*; *Sozinho*; *Amigos*; *Relaxar*; *Encrenca*)^9^. This toll consists of nine questions and seeks to identify adolescents’ risk of using psychoactive substances, aiming at prevention and early detection.
12. Questions regarding the use of a knife, physical or sexual violence, contraception.
13. Main issues diagnosed in clinical practice.

### Study population

The study population consisted of a convenience sample that included nurses from services that provide care to adolescents in the state of São Paulo. For this purpose, e-mails were requested from and authorized by the Brazilian Society of Pediatric Nurses (SOBEP) to select professionals working in São Paulo and from the Regional Council of Nursing of the State of São Paulo (COREN-SP).

### Recruitment strategies

In 2018, COREN-SP sent the questionnaire twice, in a period of thirty days, to 100,000 e-mails from professionals registered on the Council’s website, of which it is not possible to know how many returned because they were wrong or outdated. At the time of the request, the Council reported that the participation rate is usually low (1 to 5%), in a universe of more than 100,000 professionals.

SOBEP made available the mailing list with 1,541 e-mails, but more than a thousand e-mails returned because they were outdated or wrong, and each professional is responsible for updating their address.

Participants received an e-mail presenting the survey, stating that their responses would be confidential, and the response link would be sent in an individual e-mail. No incentives were offered. Reminder emails were sent periodically between August 2018 and October 2019, at fortnightly intervals.

Participants were also approached individually by the researcher at a Nursing Congress and the study objectives were explained. Then, the printed version of the questionnaire was given to professionals who claimed to work in the state of São Paulo and agreed to participate. After filling out the questionnaires, they were collected by the researcher and the answers were added to the REDCAP system. As the questionnaire does not require name or e-mail identification, data were added without compromising the study and the participants. 

### Sample size

Sample calculation was impossible since the survey was sent via e-mail and adherence was voluntary, and the answers depended on the professionals. However, statistical validation for sample relevance was conducted: for a 0.05 alpha error, with a sample of 1,617 individuals, and a 38.7% proportion (percentage of those who work with adolescents), the margin of error varied between 4.4% (for 95% power) and 3.4% (for 80% power). The exact ratio difference test for a two-tailed constant was used on the G*power software version 3.1.9.3.

### Statistical analysis

Results are presented as median (range) or mean ± standard deviation for continuous variables and number (%) for categorical variables. Nurses working with adolescents were divided into two groups based on the median length of experience working with adolescents: group A ≤ 5 years and group B > 5 years. Data in the two groups were compared using Pearson’s chi-square test or Fisher’s exact test. In all statistical tests, the significance level of the independent variable was set at 5% (p < 0.05). Analyses were performed using the statistical software SPSS v.18 for Windows. The variables with incomplete answers were identified in each table. 

### Ethical aspects

This study was approved by the Ethics Committee of Hospital das Clínicas de São Paulo under opinion number 2,296,748, and all participants signed a consent form prior to data collection.

## Results

In total, 1,632 participants answered, with a predominance of female professionals (89%), Brazilian (99%), with part-time work hours (69%), and a balance of professionals working in public or private service. Only 632 (38%) of the nurses attended to adolescents (Table 1).

The support treatment available and the characterization of the care provided by nurses are described in Table 2.

**Table 1:** Demographic data and characterization of nurses who cared for adolescents. São Paulo, SP, Brazil, 2018-2019

Variables	Nurses n=632
Demographic Data	
Womankind	568 (90)
Nationality	
Brazil	630 (99.8)
Academic Education	
Specialization	87 (13.8)
Master’s degree	22 (3.5)
Doctorate degree	16 (2.5)
More than one professional affiliation	
Yes	138 (21)
Workplace	
Public Service	309 (48.9)
Private Service	185 (29.3)
Hospital Stay	110 (17.4)
Public University	109 (17.2)
Primary Health Care Center	86 (13.6)
Private University	75 (11.9)
Work Hours n=597	
Part-time	412 (69)
Exclusive work with adolescents	
Yes	68 (11.2)
Adolescents attended/month n=606	
Less than 50 patients	405 (66.8)
From 51 to 100 patients	128 (21)
More than 100 patients	73 (12)

Results are presented in n (%)

**Table 2:** Supportive treatment available and assistance provided to adolescents. São Paulo, SP, Brazil, 2018-2019

Variables	Nurses n=632 (%)
Laboratory Test Collection	217 (34.3)
Measurement of Vital Signs	189 (30.0)
Intravenous Medication Administration	183 (29.0)
Evaluation of Anthropometric Data	166 (26.3)
Multidisciplinary Team Approach	164 (25.9)
Pain Assessment	163 (25.8)
Orientation to the Patient and Family about the Treatment	157 (24.8)
Psychological Support	151 (24.0)
Consultations with other Professionals	147 (23.3)
Electronic Medical Record	141 (22.3)
Sexual Activity Orientation	111 (18.0)
Application of Life’s Quality Questionnaires	38 (6.0)
Participation in Clinical Studies	37 (5.9)
Transition Plan for an Adult Healthcare Provider	37 (5.9)
Verification of vaccination booklet	
All queries	256 (42.7)
Twice a year	161 (26.8)
Not once	134 (22.3)
Once a year	49 (8.2)
Adolescent care separately n=604 (at least once/year)	
Yes	311 (51.5)
Use of the Instrument CRAFFT* n=605	
Unknown the instrument	424 (70.2)
Yes	146 (24.0)
No	35 (5.8)
Questioning about sexual or physical violence	
No	276 (46.0)
Questioning about the use of melee weapons/firearms n=604	
No	512 (84.8)
Adolescent Nursing Process	
Yes	351 (58.5)
Good relationship adolescent-family n=604	
No	343 (54.3)

Results are presented in n (%); *CRAFFT tool

The main issues diagnosed in clinical practice for adolescents, despite being extremely relevant in this population, were mentioned by less than a quarter of professionals (Table 3).

**Table 3:** Main issues diagnosed in clinical practice and the transition process of care for adolescents. São Paulo, SP, Brazil, 2018-2019

Variables	Nurses n=632 (%)
Main issues diagnosed in clinical practice	
Pregnancy	142 (22.5)
Problems with self-identity	120 (19.0)
Contraception	109 (17.2)
Nutrition	109 (17.2)
STD/AIDS	107 (16.9)
Problems with autonomy	106 (16.8)
Drug Addiction	96 (15.2)
Gender Identification	84 (13.3)
Need for confidentiality of information	83 (13.1)
Smoking	83 (13.1)
Homosexuality	78 (12.3)
Violence	76 (12.0)
Transition from care to adult	
Age-based transfer	97 (15.3)
There is no established program	66 (10.4)
Unknown	53 (8.4)
Transition to same-service specialist	49 (7.8)
Transition to General Practitioner in the Health System	39 (6.2)
Transition to specialist from another service	35 (5.5)

Results are presented in n (%); *CRAFFT tool

Afterwards, professionals who work with adolescents were divided into two groups according to professional experience to find possible differences in the provision of care related to the experience. The median experience of professionals was 58 (0-1500) months, and the groups were named Group A (≤60 months of experience) and Group B (>60 months of experience), as shown in Table 4.

**Table 4:** Group A (≤5 years of experience) and Group B (>5 years of experience). São Paulo, SP, Brazil, 2018-2019

Variables	Group A ≤5 years of experience N=352	Group B >5 years of experience N=280	***P***
Exclusive work with adolescents	45/339 (13)	23/268 (9)	0.071
Part-time working day	233/337 (69)	181/264 (69)	0.93
Exclusive work in the public service	120/352 (34)	104/280 (37)	0.45
Income less than R$3,000.00	73/338 (22)	23/267 (9)	<0.001
Income between R$3,000.00 and R$5,000.00	149/338 (44)	79/267 (30)	<0.001
Income between R$5,000.00 and R$7,000.00	78/338 (23)	73/267 (44)	0.26
Income between R$7,000.00 and R$10,000.00	38/338 (11)	92/267 (35)	<0.001
≤ 50 patients attended/month	232/338 (69)	169/267 (63)	0.19
Separate service (at least once/year)			
Yes	174/228 (52)	136/262 (52)	0.93
Verification of the vaccination booklet was carried out	263/334 (79)	203/265 (77)	0.55
Sexual orientation	48/352 (14)	30/280 (11)	0.33
Most recommended Contraception strategy			
Condom	83/352 (24)	71/280 (25)	0.64
Main subject diagnosed in Clinical Practice			
Pregnancy	77/352 (22)	62/280 (22)	0.92
Main service profile performed			
Treatment of patients with chronic diseases	68/352 (19)	58/280 (21)	0.69
Use of the Instrument CRAFFT*			
Yes	26/104 (25)	10/79 (13)	0.04
Unknown to CRAFFT*	233/259 (90)	184/194 (95)	0.08
Questioning about sexual or physical violence			
Yes	196/336 (58)	128/259 (49)	0.03
Questioning about the use of melee weapons/firearms			
Yes	60/335 (18)	31/263 (12)	0.04
Adolescent Nursing Process			
Yes	199/336 (59)	154/258 (60)	0.93
Good relationship adolescent-family			
Yes	132/335 (39)	125/261 (48)	0.04
Drug Addiction diagnosed in Clinical Practice	39/352 (11)	53/280 (19)	0.01
Alcohol and smoking in Clinical Practice	39/352 (11)	40/280 (14)	0.23
Performed Routine Monitoring	59/352 (17)	28/280 (10)	0.02
Age-Based Transition of Care for Adults	49/352 (14)	42/280 (15)	0.73
Indication of Work with Adolescents	326/337 (97)	252/264 (96)	0.52

Results are presented in n (%); *CRAFFT tool. São Paulo basic salary in 2018 = R$ 1,127.23; São Paulo basic salary in 2019 = R$ 1,183.33

## Discussion

This comprehensive and unprecedented work was supported by the Brazilian Society of Pediatric Nursing and the Regional Nursing Council of the state of São Paulo.

The data revealed heterogenous care, with only 38% of the nurses involved in caring for adolescents, and 11% exclusively. When compared with the demographic data of São Paulo and the contingent of adolescents, this number confirms the importance of an instrument or care protocol that is easily accessible to professionals, so that the adolescents’ demands are met in their entirety.

When the analysis was performed with professionals who care for adolescents, only 13.8% had a specialist degree, 3.5% a master’s and 2.5% a PhD. The COREN-SP^10^ website has 18,408 professionals registered as specialists, masters, and PhD’s. This number is equivalent to 9.57% of specialist professionals, but directed to adolescents, only 19 professionals are registered, and although this registration is not mandatory by the Council, the number of specialist professionals in this area is very small.

Nursing education is based on a curriculum, which can be different in many institutions and approach adolescence in non-ideal ways, the responsibility for training or improving the professional is usually accepted and often stimulated by health institutions.

Remuneration is another important aspect that impacts the performance of activities, updates, and training in the *lato sensu* and *stricto sensu* models. More than half of the professionals participating in this work receive up to R$ 5,000.00, regardless of the type of contract. The nursing category faces the lack of regulation on the remuneration, and currently fights for the establishment of the national salary floor via the approval of Bill 2,564/2020, which institutes the national salary floor and a 30-hour working week for nursing professionals. Such project intends to establish a R$ 7,315.00 salary floor.

The median age of the professionals was 38 years (22 - 74), the median working time with adolescents was 58 months (0-1500) and 48.9% of the nurses worked in the public service. These data characterize young professionals who have recently worked with adolescents. Enjoying working with this group was the main reason for choosing to work with adolescents (16%), although most of the sample has cared for less than 50 adolescents *per* month in the last year.

The nursing consultation, a mandatory work tool for carrying out the nursing process, aimed at adolescents, was reported by only 58.5% of professionals. This is relevant both are private activities of nurses carried out to contribute to the quality of patient care. Nursing consultation is a problem-solving action that requires technical, cognitive, and interpersonal skills, with systematized and interrelated actions aimed at compensating the basic needs of the patient/family/community^11^.

The nursing consultation also allows the professional to check the vaccination booklet and address topics such as sexuality, contraceptive methods, drug addiction and violence.

The analysis of the care provided showed that 42.7% of the participating professionals checked the vaccination booklet at all appointments, and the main activities performed were the collection of laboratory tests (34.3%), measurement of vital signs (30%), administration of intravenous medication (29%) and evaluation of anthropometric data (26.3%), in contrast to monitoring of medication adherence, contraceptive dispensing, application of quality of life questionnaires, transition plan to an adult health professional, all of which were performed by less than 13% of professionals.

Monitoring medication adherence is available in only 12.7% of services and is in line with a recent study carried out in Latin America^12^, where adherence to treatment and pregnancy are shown as major challenges in this population. Another study carried out with patients with pediatric autoimmune chronic rheumatic diseases describes the low adherence to medical treatment in 8/33 of the patients (24%) and shows a trend towards a correlation between socioeconomic factors and low adherence^13^. However, these studies did not use any scale to assess medication adherence, which can be very useful for nurses in diagnosing problems subject to intervention and, therefore, used in nursing consultations.

Available studies on treatment and the importance of adherence do not focus on the role of nurses and on the use of instruments that help to verify adherence, quality of life, disease acceptance, adolescent and family relationships and problems inherent to adolescence.

Adherence to drug treatment can be verified using instruments available in clinical practice, such as the Morisky and Green Test (TMG), composed of four questions that identify patients’ attitudes and behaviors regarding drug intake. The instrument has quick application and allows a simple diagnosis^14^.

In this study, 70% of professionals are not aware of the existence of the CRAFFT tool, which assesses the risk of alcohol and drug use by adolescents, helps to detect the misuse of these substances and provide targeted treatment^9^ ^,^ ^15^, revealing an important gap for appropriate treatment regardless of experience working with adolescents. 

Adolescent pregnancy was reported by 22.5% of professionals as the main issue diagnosed in clinical practice for adolescents. This result is in line with data from the World Health Organization report released on May 17^th^, 2018, which revealed the annual estimate of 12.8 million births among adolescent girls aged 15 to 19, noting that early pregnancy can increase the risks for newborns and young mothers like, also corroborating the worldwide concern about this public health problem^16^.

Questions aimed at the current Brazilian context should also be considered in the care provided to adolescents, such as the use of melee weapons or firearms, suffering from domestic or sexual violence, and the separate assistance provided to adolescents and their families as a part of all consultations. This study showed that, although more than half of these nurse’s care for adolescents (51.5%) and their guardian separately in at least one appointment a year, a considerable number of professionals did not question the adolescent about the use of firearms or melee weapons (84.8%) and did not address all these issues in the day-to-day care; 46% also did not ask about suffering from sexual or physical violence, and only 45.7% believe that adolescents have a good family relationship, which can have a negative impact on the treatment and later on the transition to adult care.

Regarding violence against adolescents, a previous study demonstrated the important role of nurses in basic health units in the notification of cases of violence against children and adolescents to the Guardianship Council. The nurses pointed out as difficulties in reporting violence against adolescents the lack of family knowledge and information and the ineffectiveness of the Guardianship Council. Professionals acted in prevention in consultations, home visits, group activities, partnerships with schools and teachers, and festive events^17^.

The transition to adult care is important and deserves attention. This study identified that only 13.3% of nurses claim to have an institutional transition to a specialist physician, showing the gap that exists in many adolescent services regarding the ideal transition, and the need for reflection on the responsibility of health services and their professionals on the best time to carry out the transition and how to properly prepare the professionals who will do so. In this sense, another research^18^ sought to understand the meaning of the experiences that adolescents with HIV/AIDS through vertical transmission attribute to the transition process of care between child and adult reference health services, and showed that they consider it a painful and difficult to accept process, especially due to the lack of knowledge about the new service and the successful change of approach to the transition, since they have already established a trusting relationship with the professionals who accompany them.

Currently, transition programs aimed at preparing adolescents as early as possible exist, developing independence and self-care (from 12 years of age), monitoring the transition process, assessing transition readiness, planning for the transition by identifying adolescents’ priorities, especially for decision-making from the age of 16 onwards, transference and integration into adult care and completion of transference^19^ ^-^ ^24^. In the absence of such program established in the institution, nurses should be aware of their existence to adapt some activities, whenever possible, and assist in this extremely important process.

Additional analyses did not reveal statistically significant differences in variables that possibly presented different results between professionals with more or less experience. On the other hand, it was observed that professionals with less experience (Group A) work more exclusively with adolescents, provide more sexual orientation and guidance on vaccination when compared to the group of more experienced professionals. Group B acted more in exclusive work in the public service, provided more care to patients with chronic diseases, provided guidance on the use of condoms as a contraception strategy, and performed more on the nursing process aimed at adolescents.

The limitations of this study include the low return of answers from professionals, even with the scheduled sending of the questionnaires for a considerable period, as verified in other studies of similar design, without compensation for the answered questionnaires, and some questionnaires with incomplete answers.

The study results show that nursing care for adolescents in São Paulo does not occur uniformly, nor does it use available tools to assess adherence and adolescent particularities and corroborates the importance of creating an adolescent care protocol to be used by nurses in this state. 

## Conclusion

The relevance of this study is justified by the scarcity of research with information on nursing care for adolescents and the importance of systematized care with a biopsychosocial approach. The data reveal that most professionals do not have a graduate degree in the area, still do not address important issues of adolescence and difficult to be addressed without prior training or preparation, but who are satisfied with the work and would recommend working with adolescents for other professionals.

We identified the need for a care protocol that can be replicated and that addresses the treatment and the particularities of adolescents, ways to improve adherence and the transition process to adult care.
